# Idiopathic Intracranial Hypertension and Multiple Sclerosis Overlap

**DOI:** 10.7759/cureus.16305

**Published:** 2021-07-10

**Authors:** Jaqueline Stoutin, Jerry Fan

**Affiliations:** 1 Internal Medicine, Baylor Scott & White Medical Center - Temple, Temple, USA

**Keywords:** idiopathic intracranial hypertension, papilledema, multiple sclerosis, headache, lumbar puncture

## Abstract

Idiopathic intracranial hypertension (IIH) and multiple sclerosis (MS) occur with a higher incidence in women of childbearing age and may be associated with other clinical entities. Both disease processes alter cerebrospinal fluid (CSF) dynamics and may present similarly with headache and visual changes. We report a case of a 33-year-old morbidly obese woman who developed progressive worsening blurry vision and unilateral temporal headache. She was found to have papilledema which prompted workup for intracranial hypertension. Her imaging and CSF findings were suggestive of a demyelinating process such as MS in addition to IIH.

## Introduction

Idiopathic intracranial hypertension (IIH) classically presents in obese females of childbearing age [1-3]. It manifests as headache associated with vomiting, pulsatile tinnitus, transient visual loss with papilledema, and diplopia due to cranial nerve IV palsy [1-5]. It occurs due to disruption in cerebral spinal fluid (CSF) equilibrium, either inadequate reabsorption of CSF or overproduction, with the etiology of the underlying mismatch not yet known [1-5]. The increasing CSF leads to increased intracranial pressure, according to the Monro-Kellie hypothesis, which damages vulnerable structures, particularly the optic nerve fiber layer [1-6]. The first-line treatment is acetazolamide along with weight loss or dural venous sinus stenting [1-2,5,7-10]. In select patients, the workup for intracranial hypertension may reveal an underlying demyelinating disease process such as multiple sclerosis (MS).

## Case presentation

A 33-year-old woman with a history of morbid obesity (385 pounds, body mass index 62.16 kg/m2), tobacco use, and anxiety and depression presented to the ophthalmology clinic with complaints of progressive blurry vision and peripheral visual field defects over the past month after stopping minocycline treatment. She noted that her visual disturbances were associated with a unilateral temporal headache and nausea without emesis. These symptoms worsened with bending and straining (Valsalva). On initial exam by an ophthalmologist, bilateral optic disc edema was found and was confirmed by optical coherence tomography of the retinal nerve fiber layer.

**Figure 1:**
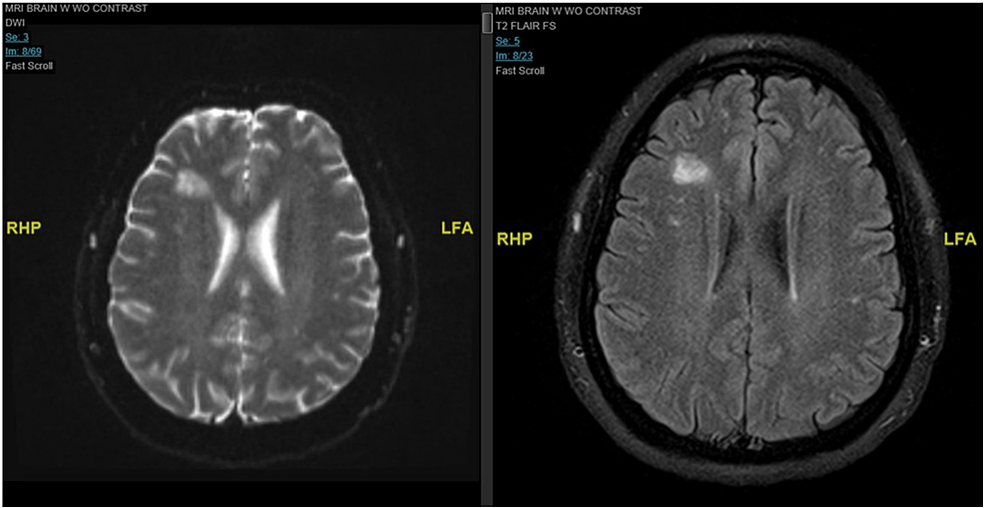
T2 DWI (left) and FLAIR MRI (right) MRI demonstrating a globular focus of increased signal intensity within the right frontal lobe juxtacortical white matter measuring 14 x 17 mm transaxially. DWI: diffusion-weighted imaging; FLAIR: fluid-attenuated inversion recovery

A lumbar puncture (LP) was performed which showed an elevated opening pressure of 37 mmH2O, confirming the presence of intracranial hypertension. Magnetic resonance imaging (MRI) of the brain showed mildly prominent fluid within the optic sheaths, suggestive of reverse cupping of the optic discs. Additionally, the MRI showed globular hyperintense lesions on T2 and FLAIR (fluid-attenuated inversion recovery) within the juxtacortical white matter, without periventricular lesions (Figure 1). These findings are consistent with elevated intracranial pressure, possibly in the setting of a concomitant demyelinating process. Magnetic resonance venography showed no evidence of occlusion or thrombosis. CSF analysis from the lumbar puncture revealed the presence of 15 oligoclonal bands and lymphocytosis. The MRI findings in conjunction with the oligoclonal bands would be suggestive of a demyelinating process such as MS in addition to IIH.

She was started on acetazolamide to help with her intracranial hypertension and referred to neurology for further evaluation of demyelinating disease. On follow up with ophthalmology one week later, her headaches and visual symptoms were much improved. Optical coherence tomography of the retinal nerve fiber layer showed improvement in optic disc edema, with remaining edema of moderate severity and gliosis of surrounding vessels with no hemorrhages. The following month, her headaches and vision remained stable, with headaches occurring about once per week with no visual field defects, but with persistent bilateral moderate optic disc edema, consistent with findings from the previous month. Three months after her initial presentation, she showed only mild signs of optic disc edema in her left eye and resolution of edema in the right eye. Given the high suspicion for multiple sclerosis from the findings on MRI and oligoclonal band findings on CSF, she is being followed closely in the Neurology Clinic for further workup of demyelinating disease.

## Discussion

Headaches are a common presenting complaint in the primary care setting [11-12]. IIH and MS can often present with overlapping symptoms such as headaches [7,9-12]. A thorough fundoscopic exam can help differentiate between IIH and other causes of headache [7,10,13-14]. Further workup is important when evaluating IIH as demyelinating diseases such as MS and neuromyelitis optica (NMO) can often overlap [12]. Imaging studies such as MRI and antibody studies such as AQ4 and MOG can be important supportive clues to a demyelinating disease. [12]

The gold standard for the diagnosis of IIH is an intracranial probe [1,9,15]. However, given the invasive nature of this procedure, the opening pressure on LP has been utilized as a less invasive diagnostic tool, with a CSF opening pressure >25 cmH2O in an obese patient or >20 cmH2O in a non-obese patient considered diagnostic of elevated intracranial hypertension [1,9,15]. Before a diagnosis of IIH can be made, secondary causes of intracranial hypertension must be evaluated [9]. In this case, the patient had overlapping elements of both IIH and MS during her workup for headache, which are outlined by the modified Dandy criteria and McDonald criteria, respectively [16-18]. A thorough workup with CSF analysis and MRI of the brain should be prompted in the setting of signs of elevated intracranial pressure. Lab studies should be used to further differentiate MS-like syndromes such as neuromyelitis optica spectrum disease marked by antibody testing against aquaporin-4 or MOG [18]. In this case, an underlying demyelinating disease was suggested by MRI findings; further testing for aquaporin 4 and MOG antibodies was deferred until follow-up with Neurology.

MS is typically found in females of childbearing age, with greater prevalence in Caucasian women and those who are vitamin D deficient [1,10-12,15]. The earliest manifestation of demyelination is typically optic neuritis, leading to decreased visual acuity or color blindness [6,10]. Optical coherence tomography can be normal as well, with findings on optical coherence tomography usually showing nasal thinning of the inner layers of the retinal nerve fiber layer [13-14]. An MRI of the brain and spine is performed along with LP to diagnose MS, with characteristic findings including oligoclonal bands and lymphocytic pleocytosis in CSF, with periventricular white matter plaques that are separated in time and space [6]. This patient’s presentation is best explained by intracranial hypertension secondary to MS. Using the revised 2017 McDonald criteria, she had elements of an MS diagnosis with findings of juxtacortical white matter lesions on MRI combined with the oligoclonal bands in CSF. The presence of oligoclonal bands is highly associated with the relapsing presentation of MS [18]. Given her young age, this may have been a first-time event, which warrants close follow-up with Neurology for future development of neurological symptoms.

It is possible that multiple sclerosis and intracranial hypertension exist on a spectrum, as both have been found to alter CSF fluid dynamics, most often through arachnoid granulations and venous stenosis [2,13-14], In patients with MS, increased area of transverse and sagittal sinuses can be seen due to outflow stenosis associated with reduced venous sinus compliance [13-14]. The area of the superior sagittal sinus has been shown to differ between IIH and MS, with no appreciable change and an increase in measurements, respectively [13-14].

For patients with intracranial hypertension symptoms refractory to medical management with acetazolamide, a developing treatment is emerging in dural venous sinus stenting [1,13-14]. This can increase the passive drainage of CSF through arachnoid granulations [1,13-14]. The success of this treatment supports the theory that increased intracranial elastance of a rigid, poorly compliant dura may play a role in the development of IIH [8]. This information provides relevant support to the interplay between MS and the development of intercranial hypertension, both of which can manifest as headache and visual disturbances. This mechanism of action of decreased venous outflow accounts for how MS leads to increased intracranial pressure.

## Conclusions

IIH and MS typically present in similar patients with overlapping complaints. In a young female patient with refractory headache and ocular complaints who is found to have papilledema, it would be prudent to do an MRI of the brain and magnetic resonance venography to characterize venous outflow, followed by LP with CSF analysis in those who have a lesion identified. An optical coherence tomography would also be useful to fully characterize any optic disc edema and identify characteristic patterns that point towards primary causes of intracranial hypertension. It is crucial to distinguish between IIH and secondary intracranial hypertension in order to properly direct management. In cases of overlapping elements of IIH and MS, it is paramount to have regular interval follow-up with Neurology for the development of neurological symptoms.
